# Key factors affecting mechanical behavior of metallic glass nanowires

**DOI:** 10.1038/srep41365

**Published:** 2017-01-30

**Authors:** Qi Zhang, Qi-Kai Li, Mo Li

**Affiliations:** 1 Nanophotonics and Optoelectronics Research Center, Qian Xuesen laboratory of Space Technology, China Academy of Space Technology, Beijing, 100094, China; 2 School of Materials Science and Engineering, Georgia Institute of Technology, Atlanta, Georgia 30332, United States; 3 State Key Laboratory for Advanced Metals and Materials, University of Science and Technology Beijing, Beijing 100083, China

## Abstract

Both strengthening and weakening trends with decreasing diameter have been observed for metallic glass nanowires, sometimes even in the samples with the same chemical composition. How to reconcile the results has reminded a puzzle. Since the detailed stress state and microstructure of metallic glass nanowires may differ from each other significantly depending on preparation, to discover the intrinsic size effect it is necessary to study metallic glass nanowires fabricated differently. Here we show the complex size effects from one such class of metallic glass nanowires prepared by casting using molecular dynamics simulations. As compared with the nanowires of the same composition prepared by other methods, the cast nanowires deform nearly homogeneously with much lower strength but better ductility; and also show strengthening in tension but weakening in compression with decreasing wire diameter. The subtle size dependence is shown to be related to the key factors including internal and surface stress state, atomic structure variation, and presence of various gradients. The complex interplay of these factors at decreasing size leads to the different deformation behaviors.

The size effect of metallic glasses (MGs) is still a controversial topic. Unlike crystalline materials, bulk MG materials possess high strength, hardness and elastic limit, but poor ductility. Therefore, making this quasi-brittle material ductile or having higher toughness is the major topic for research and development^1^,^2^,^3^,^4^,^5^,^6^,^7^,^8^, one of which is through exploitation of the size effect^7^,^8^. A number of researchers have found that when the characteristic size of MGs reduces to micro- or nano-meter scale, they exhibit good ductility, even with flow-like deformation^3^,^4^,^5^,^6^,^7^,^8^. But how the strength changes with size is still perplexing. Up to now, both decrease^9^,^10^ and increase^8^,^11^,^12^,^13^,^14^ in strength are observed when the size decreases. For example, Lee, *et al*.^11^ and Lai, *et al*.^12^ reported 80% higher compressive yield strength in MG nanowires made from focused ion beam(FIB) milling compared to their bulk counterparts. With the same preparation method but different compression tests method, Volkert, *et al*.^9^ observed lower compressive yield strength than the bulk sample. For MG nanowires made via drawing, Nakayama *et al*.^10^ measured Young’s modulus and tensile strength that are below the bulk values. In addition, there are also reports^15^,^16^ of no remarkable change of strength with decreasing size.

The inconsistency may stem from multiple sources. Small size MGs made via different processes may have different atomic structures, leading to different mechanical behavior^7^,^17^,^18^,^19^. Magagnosc, *et al*.^17^ reported that the structural change induced by irradiation from focused ion beam made brittle MG nanowires ductile. Chen, *et al*.^7^ found that the extent of ductility for MG nanowires is closely related to the fabricating method. The simulation by Shi^18^ also shows that the deformation behavior of the MG nanowires is affected by the preparation method. Besides, experimental conditions could also lead to systematic error. For example, the taper of the nanowires fabricated by FIB milling would affect the mechanical behavior significantly such that the experimental data must be amended artificially to characterize the mechanical properties of the nanowires^9^,^10^,^11^,^12^,^13^,^16^,^20^,^21^.

Since MGs have no particular extended structural defects, explanation of size effect becomes a challenge without both the conceptual and technical convenience readily available for dislocations or grain boundaries often seen in crystals^22^,^23^. To understand the intrinsic size effect of MGs, it is necessary to perform detailed study, including atomistic modeling, on the premise classifying the nanowires according to the processing method. Computer simulation has the capability to discover atomic scale mechanisms and processes of size effects while avoid many complications such as the geometrical imperfection and processing defects. Although under much simplified conditions, the simulations can help us to identify the general trend in mechanical properties and various factors affecting them. Our previous simulation study on FIB MG nanowires indicates that the mechanical behavior of the nanowires below 50 nm in diameter is heavily influenced by the combination of *surface stress and internal stress*^24^. Our subsequent investigation reveals that the surface and surface related properties can change under different processing conditions^19^. The key question follows naturally: in the absence of extended structural defects, whether these two factors are still the fundamental entities responsible for the size effect in MG and are applicable to other MG nanowires. Through comparative study with the known results from FIB prepared by other methods, we expect to answer these questions.

In this work, we will investigate the size effect of the MG nanowires prepared by the procedure mimicking casting liquid into glass nanowires through molecular dynamics (MD) simulations. As we elaborate below, the cast nanowires are chosen to be studied here because they exhibit very different mechanical behavior compared with the FIB nanowires. The systematic study is, therefore, expected to shed light on the deformation mechanisms in metallic glass nanowires in general whose response is sensitively dependent on the subtle structural change and internal and surface stress. Our results demonstrate that deformation of incredible ductile cast nanowire with much loose atomic packing and gradients of density and chemical composition is still governed by the similar mechanisms found in FIB nanowires, that is, the interplay between the internal and surface stress. We show that the quantitative difference in the stresses and structural properties in the two extreme cases, FIB and cast wires, lead to significantly different mechanical behaviors. For example, strengthening is found under tension at decreasing wire size in cast wires but absent in FIB wires; and the same failure mechanism observed in cast wires initiates from the interior when under compression but from surface when under tension, despite the presence of complex gradients and subtle structural changes. Besides these highlights, we shall present more detailed results below.

## Simulation Methods

The composition of the metallic glasses used here is Cu_64_Zr_36_ for its good glass forming ability^25^,^26^,^27^ and the relatively mature interatomic potentials tested extensively in the past^28^. The samples are prepared by quenching liquid nanowires to room temperature using boundary constraint method^29^. We first take out a liquid nanowire from equilibrium bulk liquid sample and put it into a cylindrical mold with the walls having the same diameter and length as in the liquid wires. The initial aspect ratio of length to diameter for the liquid wires is 3 and the diameter is from 4 nm to 32 nm. During cooling, the location of the axes for the mold is fixed but the walls are allowed to fluctuate to release the internal stresses of the systems and adapt to the volume change related to the decreasing temperature. The constraint walls are modeled as rigid bodies, and the wall-wire surface interactions are purely repulsive via an interaction, *V*(*r*) = *K*(*r*−*R*)^3^/3; where *r* is the distance from the atom to the axis of the wall, *R* is the radius of the wall and here we use *K* = 100 eV/Å^3^. The pressure is kept at zero through the simulation via the Andersen barostat and the temperature is controlled by Nosé-Hoover thermostat. The cooling rate is 1 K/ps. Each MD time step is 10^−3^ ps. Periodic boundary condition is used along the axial direction while free boundary conditions along the other two directions. After cooling the liquid down to 300 K, the mold is removed and the nanowires are relaxed until the energy does not change within the timescale in the simulation, typically over a million MD steps. Then uniaxial tensile and compressive tests are conducted on the nanowires at a strain rate of 10^8^s^−1^. Although high compared with the ones used in experiment, especially under quasistatic loading, the strain rate is proper in the MD simulation of solid mechanical behaviors in the metallic glasses which are quenched much faster from liquid than the experimental ones. Otherwise, under slower strain rate the metastable glassy sample would exhibit continuous relaxation or viscous behaviors, rendering the simulation result unreliable.

## Results and Discussions

### Synopsis of mechanical behaviors

**Figure 1:**
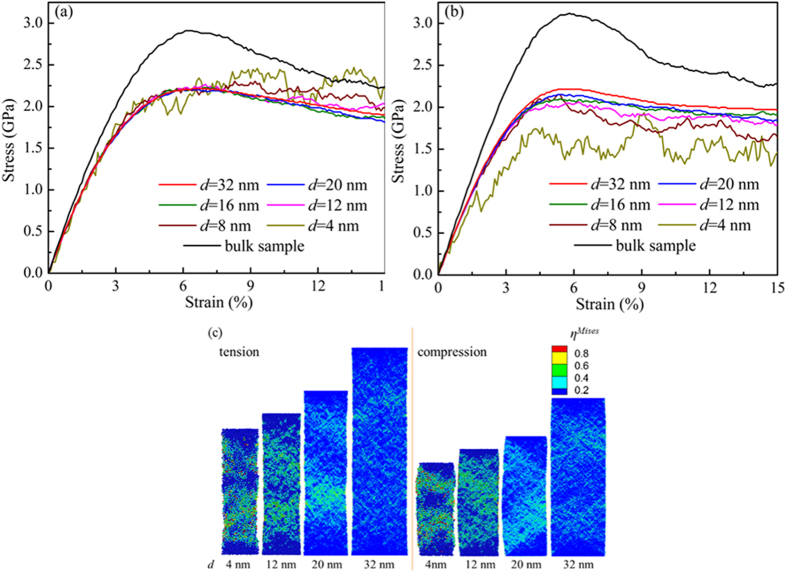
The stress-strain curves for (**a**) tension and (**b**) compression of the cast MG nanowires. (**c**) Snapshots of atomic strain in the cast nanowires with different diameters *d* (4, 12, 20, 32 nm). The local strain is colored by the magnitude of the atomic von Mises shear strain *η*^*Mises*^ at 12% overall sample strain.

Figure 1(a) and (b) show the stress-strain curves for the cast nanowires as well as the bulk sample under tension and compression. The strength of the metallic glass is defined by the yield and the maximum stress. The former is obtained by the 0.2% offset strain method and the latter is the highest stress value in the stress-strain curve. Plasticity, on the other hand, is defined by two parameters, one is the range of the strain from the yield point to the maximum stress and the other, a more practical one, is how rapid the maximum stress drops after reaching the highest value. In experiment, the rapid drop usually leads to fracture, or end of plastic deformation; but in simulation, due to the high strain rate fracture cannot be fully captured. Instead, one sees a drop of the maximum stress in the stress-strain curve. This trend is a useful indicator for plasticity. For the cast wires, the overall strength measured by the maximum and yield stresses for the nanowires is lower than that for the bulk sample. But the ductility for the wires is improved as seen by very little decline of the stress after it reaches the maximum value in Fig. 1. The plastic strain range from the yield to maximum stress can also be seen extended significantly as compared with the bulk sample, especially for the nanowires with diameter less than 10 nm. For tension, when the diameter is larger than 10 nm, the nanowires with different sizes exhibit almost similar mechanical behavior in elastic and also plastic regime around the yielding and the maximum stress as the size varies, but differ very much from each other in compression. In both tension and compression loading modes, the fluctuation in the stress-strain curves becomes more intensified as the wire diameter reduces, especially below 10 nm.

Another gauge for plasticity change is to look at the so-called atomic strains, the local deformation associated with each atom. By mapping the atomic shear strain^30^ of the atoms in the wires, we find the cast nanowires deform nearly homogeneously although certain local regions have larger plastic strain in the late plastic range (Fig. 1(c)). However, no shear band-like local deformation emerges throughout the entire loading process. The uniform distribution of the local strains indicates less tendency for brittleness, or better plasticity, which corroborates with the results seen in the stress-strain curves.

The above results show that the cast nanowires are in general ductile with homogenous deformation, or with absence of shear localization, when the diameter of the wire is smaller than 32 nm, which differs from the nanowires made via other processing routes such as FIB or high temperature impressing^19^. For the latter, brittleness and strong shear localization are the dominant features in the mechanical properties^24^.

### Residual internal and surface stress, surface thickness and property gradients

Surface in metallic glass nanowires plays a key role since the other extended structural defects are absent. We found that the surface is the source for surface stress which in turn induces internal residual stress inside the wires^24^. If no other structural or chemical imperfections present, as in the case of our atomistic simulation, these stresses could alter the mechanical behavior of metallic glass wires in significant ways, especially when the sample size is small.

**Figure 2:**
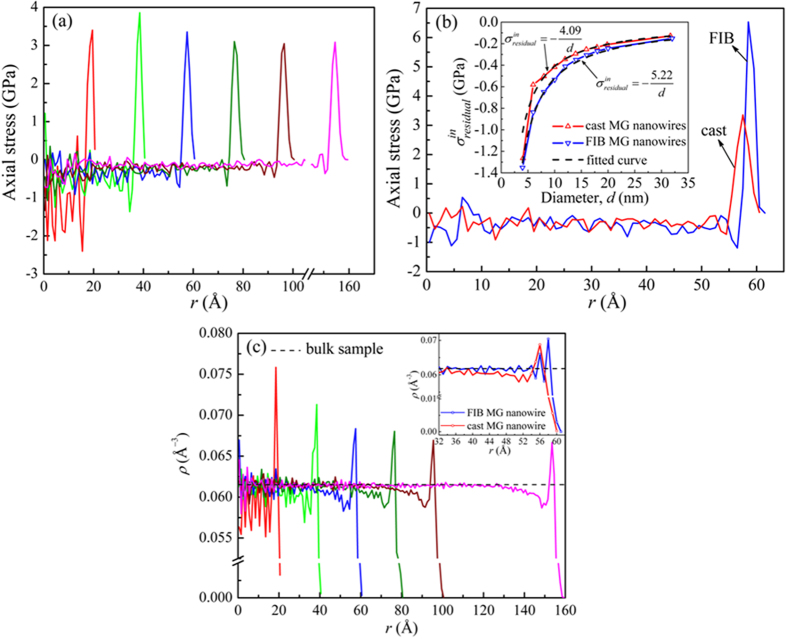
(**a**) The axial stress profiles along radial direction for cast nanowires with different diameters. (**b**) The axial stress profiles of the FIB and the cast wires with diameter of 12 nm. The inset is the Yong-Laplace relation for the internal stresses versus the wire diameter. (**c**) The density profiles along the radial direction for cast wires with different diameters. For comparison, in the inset we plot the same quantity for the FIB and the cast wires with diameter of 12 nm.

Same as observed in FIB nanowires^24^, surface and surface induced internal stresses are present in cast nanowires, but with a few noted variations. During casting, the glass forming liquid inside the mold experiences an oscillatory repulsion by the mold as the volume of the liquid inside fluctuates during cooling, as if “kneaded” by the mold. This particular quenching scheme leads to more thorough relaxation in the cast MG nanowires. In other words, the atoms could be in more stable configurations. Figure 2(a) shows the profile of the residual stress and surface stress in the cast nanowires with different diameters. The shape of the profile does not change much when the size decreases. As compared with the FIB samples, the surface residual stress is much smaller in cast wires and so is the internal stress as shown in Fig. 2(b). By fitting the internal stress (the inset of Fig. 2(b)) versus the wire diameter *d* with the Young-Laplace relation 
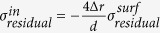
24, we find that the surface residual stress 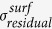 for the cast wires is only about 40% of that for the FIB samples, or 1.02 GPa versus 2.61 GPa. The residual stress inside the sample is therefore small as shown by the Young-Laplace relation. Here, Δ*r* is the surface thickness of the nanowires determined by the “affected zone” on the surface whose stresses deviate from the inside significantly according to the stress profiles (Fig. 2(a)). The thorough relaxation in the cast wires is also reflected in the “affected zone” or surface effective thickness Δ*r*: It is much broader, about 1 nm, for the cast nanowires but 0.5 nm for the FIB nanowires that can be seen in Fig. 2(b).

The presence of the surface and internal stress for the cast wires during cooling causes atomic diffusion starting from the surface region and gradually moving inward, as evidenced by Cu concentration gradient, as well as both structure and property gradients along radial direction of the wires^19^. For example, the local density gradient appears as shown in Fig. 2(c) along with chemical segregation^31^ and free volumes^19^. For comparison, in the insets of Fig. 2(c) we plot the density profiles for the cast and FIB wires. For the latter, the range of density change is much narrower as limited only in the surface region with a much small penetration depth into the wires.

### Analysis of the mechanical behaviors

**Figure 3:**
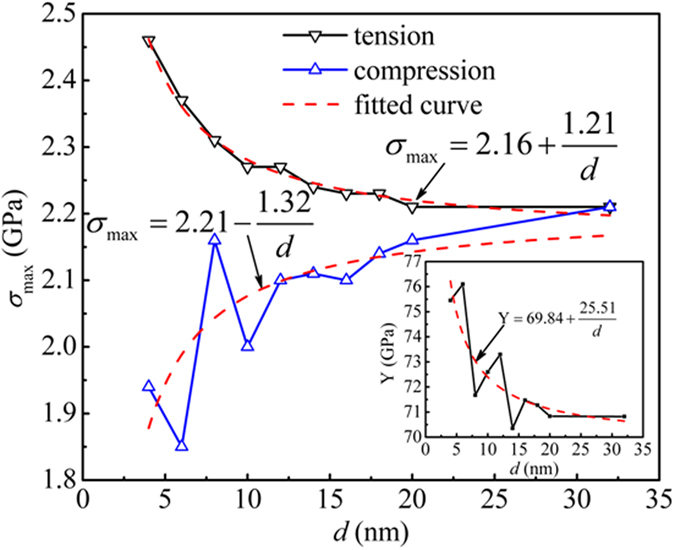
The maximum stress of the cast nanowires versus diameter d for tension and compression. The inset is the Young’s modulus versus diameter. The dashed lines are from the fitting using the relations, *y* = *A*±*B*/*d*^*n*^ shown in the figure.

From the stress-strain relations in Fig. 1, we extract the average mechanical behaviors versus the wire diameter. Figure 3 shows the maximum stress for the cast MG nanowires versus wire diameter. In tension, obvious strengthening effect is seen when the size decreases, while opposite effect, weakening, is seen in compression. We fit the data using an inverse relation, *y* = *A* ± *B*/*d*^*n*^, where *A, B* and *n* are free parameters to be fitted from the simulation results. *y* is either the maximum stress or Young’s modulus. The fitted parameters B for the maximum stresses show the change of the strength with decreasing diameter in compression is about 10% larger than that in tension, albeit in decreasing magnitude (Fig. 3): it is 1.21 GPa for tension and 1.32 GPa for compression. In other words, the size effect is more sensitively manifested in compression than in tension. And the Young’s modulus increases with reduced diameter. As comparison, FIB wires show weakening effect in both tension and compression in both the maximum stresses and modulus^24^. In the following, we shall analyze these results in terms of the surface and internal stresses, property gradient and atomic structures.

Same as in FIB nanowires, the cast wires can be considered as composed of two parts, a surface layer and an interior part or core, distinguished by their different stress state shown in Fig. 2(a). Due to the geometry, they can be considered as connected in parallel. Assuming that the wire is loaded axially and in mechanical equilibrium, the effective stress *σ*_*eff*_ of a nanowire under any applied stress is the summation of the surface *σ*_*surf*_ and the interior ones *σ*_*core*_ weighted by their respective volume fractions, *x*_*surf*_ and *x*_*core*_, *σ*_*eff*_  = *x*_*core*_*σ*_*core*_ + *x*_*surf*_*σ*_*surf*_^24^. Both *σ*_*surf*_ and *σ*_*core*_ can be obtained separately from the simulation as shown below.

**Figure 4:**
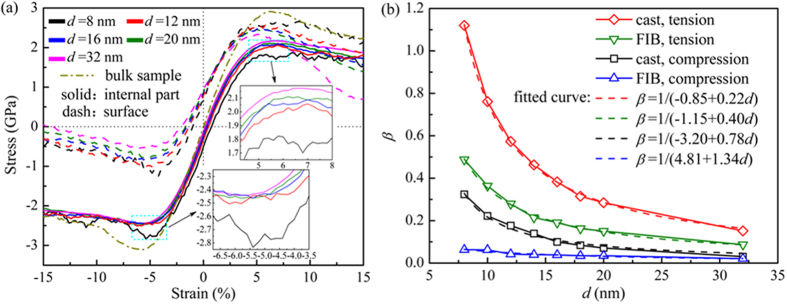
(**a**) The stress-strain relations for the surface and interior of the cast nanowires with different diameters under tension (positive strain) and compression (negative strain). The insets are the enlarged views at the corresponding maximum stress for the interior region. For comparison, the stress-strain relation for bulk sample is also shown. (**b**) The relative contribution of the surface stress against the core versus the wire diameter for cast and FIB wires.

Figure 4(a) shows the stress-strain curves for the surface and core, *σ*_*surf*_ and *σ*_*core*_. They are obtained by summing over the atomic stresses of the atoms in the surface and interior regions separately^24^. The relative volume fraction of the surface and core regions, *x*_*surf*_ and *x*_*core*_, can also be estimated based on the fact that the surface region remains stationary with a constant thickness *δ* defined by the surface stress profile (See Fig. 2), that is, *x*_*core*_ = (1−2*δ*/*d*)^2^ and *x*_*surf*_  = 1−*x*_*core*_. For the cast wires, *δ* = 1 nm and 0.5 for the FIB wires. Using these data, we can obtain the relative volume fraction of the surface and the core. *d*~7 nm, for example, is a “break” point where *x*_*surf*_  ≈ *x*_*core*_. When *d* smaller than 7 nm, the surface has larger volume fraction than the core.

Therefore, from *σ*_*surf*_ and *σ*_*core*_ and *x*_*surf*_ and *x*_*core*_, we can estimate the relative contribution of the surface and the core regions to the total stress of the wire, i.e. *β* = *x*_*surf* _*σ*_*surf*_ /*x*_*core* _*σ*_*core*_. Figure 4(b) plots *β* for the maximum stress for tension and compression as the function of the wire diameter *d*. As a comparison, we also plotted the results for FIB wires. In both cases, the surface contribution in tension is larger than compression; and cast wires have far large influence from the surface than FIB wires. For example, in tension the surface contribution of the cast wires goes from about 18% at the diameter of 32 nm to 80% at slightly below 10 nm; while only from 10 to 35% for FIB wires. In compression, it goes from about 2 to 22% for cast wires and from 1 to 5% for FIB wires. Moreover, in cast wires, the surface contribution becomes even larger than that from the core in tension when the diameter goes below 15 nm; in compression, the surface contributes about the same as the core when the “break” point, i.e. 7 nm, is reached.

As shown in Fig. 3, the maximum stress increases with decreasing *d* in tension, whereas decreases in compression, which is another subtle effect of size dependence in metallic glass nanowires – in FIB wires both decrease. The explanation of this difference rests on how the surface and core participate in mechanical deformation. During uniaxial loading, the strain remains the same for surface and the core as constrained by the surface-and-core geometry. Therefore, the maximum strength, or failure stress to be precise, is related directly to how the core or surface withstands the largest stress individually. In other words, *the failure of a wire is determined by the local stress of either the core or the surface whenever their respective maximum strength is reached first*. As shown in Fig. 4(a), surface stress is already under tension in the initial unloaded state and reaches the maximum stress first subsequently under external tensile loading under the combined initial and applied tension. Therefore, the surface dominates the tensile failure and the overall strength of the wire under tension as the maximum stress is terminated when (surface) failure occurs. Since the surface contribution to the overall strength varies with the diameter (see Fig. 4(b)) and is the largest in tension, the effective tensile stress applied to the wires needed to reach the maximum stress is less. This may explain why, when the wire diameter becomes smaller, the strength increases in tension is smaller than that in compression as seen in Fig. 3.

There is another reason for the increasing strength in tension that is related to the reduction of brittle failure at surface due to the nanowire surface state. For smaller wires (*d* < 15 nm), more than 25% of the atoms are on the surface and these fraction of atoms have more free volumes or lower atomic density close to the surface exceeds 50% as shown by the density gradient (Fig. 2(c)). Therefore, these atoms can move relatively easily on and close to the surface. We hypothesize that due to this peculiar surface state, the surface atoms are more mobile than those in the core; at increasing tension, they may behave like liquid than solid: one can imagine that if there is any local region that deforms more and develop into a local crack or necking, the mobile surface atoms would move to the region, diminishing the local deformation. Therefore, under tension the nanowires have less tendency for localization. The extra stability of the wires provides an opportunity for further loading and thus allows for the wire to reach high stress. The strengthening mechanism in small cast wires under tension is, therefore, different from the maximum stress bearing mechanism mentioned above; it is related to the enhancement of plasticity by reduction of deformation localization on the surface. For larger wires (*d* > 15 nm), for example, *d* = 32 nm, the surface contribution gets smaller as the fraction of surface atoms is smaller; and more importantly, the surface atoms start to behave more solid-like, or have the tendency for localization when a surface deformation initiates. This is shown by an abrupt drop of the surface stress at the maximum stress which resembles that in a brittle failure (see Fig. 4(a)). Therefore, for larger cast nanowires, surface acts like a defect that initiates the failure and thus determines the maximum strength.

Next, we explain the mechanical behavior under compression. Similar to tension, surface stress *σ*_*surf*_ of the wires under compression is under tensile initially (Fig. 4(a)). The initial surface tension stabilizes the surface by counterbalancing the applied compressive stress. In other words, the net (compressive) stress on the surface is smaller. Under applied compression, the surface stress remains tensile till about 2% overall strain before becoming compressive but with a small value in the range of 0.5–1 GPa (see Fig. 4(a)). On the other hand, the internal stress in the core region, *σ*_*core*_, is compressive initially and increases with increasing applied compression. But when the maximum stress is reached, *σ*_*core*_ levels off and remains relatively stationary with the size change when the diameter exceeds 10 nm (Fig. 4(a)). As compared to the surface stress, the internal stress is several times larger. Thus the internal part of the wire bears the maximum stress first and determines the failure mechanism of the wires while the surface plays no direct role. The clear evidence for the above analysis can be seen even in the wire with *d* = 8 nm under compression - the maximum stress for the interior regions drops abruptly at the maximum stress (inset of Fig. 4(a)) while the surface stress remains small and shows little change.

Finally by comparing the cast and FIB wires, we could see how sensitive the mechanical properties are to the subtle change in the few factors identified above: The residual surface stress in cast wires is small as compared with that of the FIB wires (Fig. 2). The latter have large initial tensile surface stress that remains tensile throughout compression. Also because of the relatively thin surface layer, the surface contribution in the FIB wires is small (Fig. 4(b)). These factors give rise to very different mechanical behaviors for the FIB wires, i.e. brittle and strong, as compared to the cast wires.

### Atomic packing and its effects on mechanical behaviors

The cast wires are in general weak in strength and have better ductility as compared with other MG wires prepared differently^19^. As we see above, the effects from surface and internal stress, as well as the gradients, on these mechanical properties are achieved mainly by changing the occurrence of the yielding and maximum strength and ductility. This is done through the maximum load bearing mechanism. There are other factors that may attribute to the change of the magnitude of the strength through different mechanisms, including free volume and icosahedral clusters.

It is known that the higher free volume concentration, the higher the ductility but lower the strength. The initial free volume in cast wires is about 1.25% higher than that in FIB wires^19^. The increase of free volume in case wire is caused by rapid cooling of the liquid in the mold and more importantly, the repulsive interaction of the metallic glass with the wall of the mold. The relatively high amount of free volume increase in cast wires is comparable to that caused by irradiation^17^, and thus can make the cast wires more ductile.

**Figure 5:**
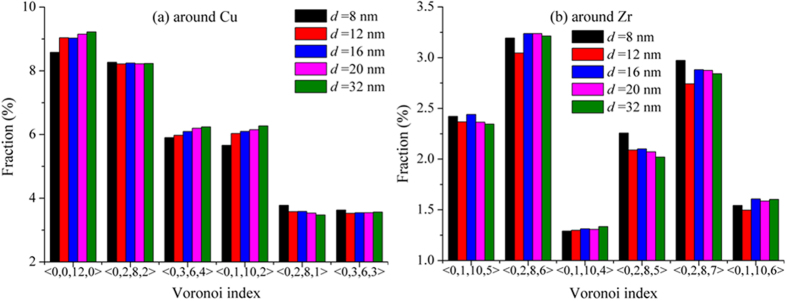
The fraction of the most popular clusters in cast nanowires with different diameters for (**a**) Cu centered and (**b**) Zr centered clusters.

Atomic structure also contributes to the mechanical properties of metallic glasses. The more densely packed local atomic clusters there are, the smaller free volume and the stronger or harder are the metallic glasses. The icosahedral clusters have long been thought to belong to this category of densely packed local structures. Its presence is thought to enhance the strength but reduce ductility^32^. Figure 5 shows the population of a few Cu- and Zr-centered local cluster packing in the coast nanowires with different diameters. In Cu_64_Zr_36_ metallic glass, due to atomic size difference, Cu atom with a smaller diameter tends to have 12 nearest neighbors of Zr and Cu that possess icosahedral packing (Fig. 5(a)), while the bigger Zr atom does not (Fig. 5(b)). Among the Cu-centered clusters, the 12 nearest neighbor cluster, or 〈0,0,12,0〉 type of cluster with icosahedral symmetry, is the most popular and several other 12 nearest neighbor clusters with partial five-fold symmetry have smaller population (Fig. 5(a)). But as compared with the nanowires prepared by other methods, the fraction of the icosahedral cluster in cast wires is much less, about more than 20% less as compared with FIB wires^19^. The significant reduction of the icosahedral cluster population is the indication of the enhanced plasticity, and of course, reduced strength in cast wires as we reported above.

Another intriguing finding is that during deformation the 〈0,0,12,0〉 cluster population does not change as much as that in the wires processed by other methods^19^. Previous works have found that mechanical deformation in general breaks the densely packing cluster, resulting in reduction of its population and weakening of the samples. The invariance of the icosahedral cluster in cast wires suggests that the so-called densely packed local atomic packing is not participating in strengthening process as it should be. This could happen in two scenarios: One is that the icosahedral clusters are isolated, or surrounded by other clusters which can deform more easily, so leaving the former alone. Another is the icosahedral clusters themselves in the cast wires are changing but dynamically recover quickly, resulting in a steady state population. The possibilities are currently under investigation.

More intriguing is that reduction in wire size seems to lead to reduction in the cluster populations, although only about 7% from 32 to 8 nm (Fig. 5(a)). The decrease of the 〈0,0,12,0〉 cluster is correlated well with the observation that the smaller wires are more ductile. Once again, we can link the reduction of the icosahedral clusters to the “kneading” of the liquid during cooling by the vibrating hard mold wall. The repeated motion of the mold wall exerts repeated tensile and compressive pressure along the wire radial direction. The smaller the wire diameter, the more effective the kneading, that prevents the cluster from forming during cooling.

## Conclusions

Compared with nanowires prepared by other methods, cast MG nanowires exhibit strengthening in tension but weakening in compression with the reduction of wire diameter. We identified several key factors that affect the size effect on the mechanical properties of metallic glass nanowires. By comparing with the FIB wire, we are able to clarify how these factors vary as a result of sample preparation and how they affect the overall mechanical properties of MG nanowires. We show that surface and internal stresses play a fundamental role in determining the strength and failure of the nanowires by controlling the occurrence of the yielding, maximum stress and fracture, which is done through the balancing of the surface and core contributions to the overall stress of the wires. We call it the *maximum load bearing* mechanism in which the maximum stress or failure is determined by either the surface or the core depending on where the maximum stress is first reached. In cast wires under tension, it is the surface with the maximum stress reached and thus the surface acts effectively as a defect. But under compression, surface is pacified while the core experiences the maximum stress first.

We found that the surface induced internal residual stress follow Young-Laplace relation. The key parameters in this relation is the surface stress and the surface thickness. As compared with other wires synthesized via different routes such as FIB, the cast wires have a wider or more diffuse surface layer with low surface and internal residual stresses. These factors, along with the combined effect of the applied stress, determine the overall stress and strain behaviors in the wires through the maximum load bearing mechanism. One of the subtle consequences is the strengthening in tension but weakening in compression, while strong, but brittle behaviors are found in FIB processed metallic glass nanowires with narrow surface thickness and high surface stress.

Besides the surface effects, high free volume and low dense atomic local packing population are observed in cast nanowires. As compared with the surface stress and related factors, these quantities play an important but different role in the mechanical properties by changing the overall strength values. In cast wires, the high free volume and low population of the icosahedral clusters are responsible for reducing the strength reduction and enhancing the ductility.

As we demonstrated here, it is not only difficult but complex to analyze the mechanical properties of metallic glass nanowires without the convenience of relying on the extended structural defects as in crystalline materials. The first is to find and identify the relevant factors, including possible defects. The factors we have found and identified so far are much different from those from crystal dislocations and other extended structural in crystals. Second, It seems to us so far that there is no single factor that dominates the outcome. We show that it is much complicated to find the net effect originated from the sample size from a plethora of factors and their complex interplay that collectively lead to the overall mechanical response in metallic glass nanowires. Therefore, in experiment and also modeling, one must take great caution in clarifying all related factors that could result from many sources such as preparation method, surface and interior condition, and atomic structure. Surface and surface induced internal stress are two key factors as identified in this simulation albeit at small wire sizes. In real experiment, one faces more challenges including identifying various sample imperfections and chemical and structural heterogeneities. These factors may also contribute significantly to the change of mechanical properties of the MG nanowires.

The authors like to acknowledge the financial support of this work by the National Thousand Talents Program of China.

## Additional Information

**How to cite this article**: Zhang, Q. *et al*. Key factors affecting mechanical behavior of metallic glass nanowires. *Sci. Rep.* **7**, 41365; doi: 10.1038/srep41365 (2017).

**Publisher's note:** Springer Nature remains neutral with regard to jurisdictional claims in published maps and institutional affiliations.
